# Take a Break for Memory Sake! Effects of Short Physical Activity Breaks on Inhibitory Control, Episodic Memory, and Event-Related Potentials in Children

**DOI:** 10.3390/brainsci14070626

**Published:** 2024-06-23

**Authors:** Eric S. Drollette, Praveen A. Pasupathi, Alexis B. Slutsky-Ganesh, Jennifer L. Etnier

**Affiliations:** Department of Kinesiology, University of North Carolina Greensboro, Greensboro, NC 27412, USA; papasupathi@uncg.edu (P.A.P.); abslutsk@uncg.edu (A.B.S.-G.); jletnier@uncg.edu (J.L.E.)

**Keywords:** children, physical activity, cognition

## Abstract

The pervasive sedentary lifestyle exacerbated by the COVID-19 pandemic has significantly reduced physical activity (PA) among school-age children, necessitating innovative strategies to evaluate short PA breaks that are feasible in a classroom setting. This study explored the cognitive and neurophysiological (electroencephalography; EEG) impacts of short bouts of different PA modalities on inhibitory control (flanker task) and episodic memory (word recognition task) in children. Utilizing a within-participants cross-over design, thirty-six children (*n* = 36; 9–12 years old) attended the lab on three separate days with each visit including either a 9 min bout of sustained moderate-intensity cycling, high-intensity interval exercise (HIIE), or seated rest. Event-related potentials (ERPs) were assessed during the flanker task (P3 component) and the word recognition task (LPC and FN400 components) to elucidate the neural mechanisms underpinning behavioral outcomes. Findings indicated no differences in flanker performance but greater episodic memory recall for HIIE compared to seated rest. Neurophysiological results revealed no differences for P3, but notably larger amplitude for LPC and FN400 postcycling, particularly over parietal electrode sites. These results underscore the potential of short PA breaks to improve cognitive and neurocognitive function in children, offering a feasible integration strategy into daily school routines without extensive time commitment.

## 1. Introduction

In response to the COVID-19 pandemic, governments worldwide imposed social distancing measures affecting 1.5 billion school-age youths [1]. This led to a surge in sedentary screen time [2] and curtailed opportunities for physical activity (PA) at school (e.g., recess, physical education, and active lessons) and in the community (e.g., recreational sports, clubs, and fitness facilities). Drastic reductions in total daily PA (~17 min) occurred from before to during the pandemic [3], exacerbating an already ongoing decline in PA among children [4,5]. Such trends jeopardize both physical and psychological functioning especially as students transition back to the classrooms postpandemic. As such, it may be necessary to investigate innovative opportunities within a regular school day to increase PA for both psychological and physical health in children. Research is well documented regarding the positive effects of PA on brain and cognitive function in children [6]. Notably, even single bouts of PA have demonstrated significant benefits [7,8,9,10,11,12,13,14,15]. However, most of these studies in children included prolonged (i.e., greater than 20 min) and sustained moderate-intensity PA, which may not be feasible in a classroom or school setting. Recognizing this gap, our study aimed to examine the effects of classroom-friendly short PA bouts on children’s brain function and cognition.

Building upon the importance of PA in academic settings, it is crucial to delineate specific cognitive processes bolstered by acute bouts of PA. Researchers have underscored certain cognitive components, notably inhibitory control and memory function, as pivotal for academic achievement [16,17,18]. Inhibitory control is part of executive function and involves the ability to inhibit a prepotent action in response to the current stimulus and to refrain from irrelevant events [19]. Extensive research provides consistent evidence revealing that acute PA positively influences inhibitory control to a greater degree compared to other executive function outcomes [14,20,21,22]. In contrast, memory, particularly episodic memory—which enables individuals to recall episodes related to prior spatial and temporal events [23]—has garnered less empirical attention in the context of acute PA in children. However, extant studies corroborate the enhancing effects of acute PA on this cognitive component [12,24,25,26]. For example, recent evidence from our lab evaluating light–moderate-intensity walking (~26 min duration) revealed improved recall for primacy words (i.e., the first ten words in the word list) after exercise conditions compared to seated rest [12].

The above-mentioned results are promising and further suggest that providing PA breaks for children may improve underlying cognitive processes that facilitate academic outcomes. However, a significant limitation of these studies is the reliance on prolonged PA duration of 20 to 30 min. Although these PA bouts align with general health recommendations [27], such extended periods are infeasible in a classroom or school setting given inherent space and temporal limitations. There is a clear need to investigate the efficacy of shorter and more feasible PA modalities that extend into a classroom setting but maintain improvements in cognitive function. Short bouts of high-intensity interval exercise (HIIE) may be a suitable and pragmatic alternative to these constraints. HIIE constitutes short high-intensity PA in combination with low-intensity rest periods [28]. Previous research examining the influence of HIIE shows promising results on measures of inhibitory control in adults [29,30,31,32]. For instance, young adults elicited greater performance on behavioral measures of inhibitory control following 9 min of aerobic HIIE (i.e., running on a treadmill) compared to seated rest [30]. These findings are consonant with other studies exploring the effects of similar exercise modes on inhibitory control within the adult population [29,32]. Similarly, a recent meta-analysis reported the greatest effects (Cohen’s d = 0.54) on episodic memory following high-intensity PA in young adults [33]. Building on this research evidence, the present investigation sought to extend this research and evaluate the effects of concise PA (9 min) on children’s inhibitory control and memory. By exploring the efficacy of these short bouts of PA interventions, we aimed to provide a scalable and practical solution that could be easily integrated into the daily routine of schools. This study not only sought to validate the cognitive benefits of HIIE in a novel context, but also to contribute to a growing body of evidence that could impact PA guidelines within educational systems.

Given the promising implications of HIIE for enhancing cognitive functions within the practical constraints of school environments, it becomes essential to understand not only the behavioral outcomes but also the neural mechanisms underlying these improvements. To achieve this, the present study utilized electroencephalography (EEG) measures of event-related potentials (ERPs) to evaluate temporal brain function outcomes following exercise. Among the ERPs, the P3 ERP is a positive-going component occurring around 300 ms following stimulus onset. The P3 amplitude is an index reflecting the amount of attentional resource allocation during stimulus engagement and latency [34,35]. Previous research involving children revealed a larger P3 amplitude following acute PA (i.e., 20–30 min) suggesting greater attentional resource allocation in the context of inhibitory tasks [21,36]. Similarly, episodic memory is evaluated at a neural level using ERPs (frontal negative 400 or FN400; late positive component or LPC) that help distinguish distinct memory processes associated with familiarity and recollection in recognition of information [37]. Specifically, the FN400 is a negative deflection occurring between 300 to 500 ms revealing greater amplitude across mid-frontal sites suggesting familiarity-based recognition (i.e., the feeling of knowing or strength of uncertainty to an item) of new/similar items [37,38,39]. The LPC is a positive deflection between 500 to 800 ms across parietal sites and is associated with recollection (i.e., conscious, concrete, and accurate recalling of details), especially for old/studied information [38,40]. The present study sought to evaluate the impact of PA on these neurocognitive domains by examining changes in P3, FN400, and LPC.

The limited availability of PA opportunities with schools [41,42], coupled with the challenges of aligning with public health recommendations (i.e., ACSM or WHO), underscore the need for short bouts of PA opportunities in educational settings. This study sought to provide preliminary evidence for short bouts of PA that can be mimicked in a classroom setting, potentially incorporating them as “active breaks” between classes. The present study aimed to evaluate short bouts (9 min) of PA including moderate-intensity stationary cycling and high-intensity interval exercise (HIIE) in children on inhibitory control and episodic memory and underlying ERP components of P3, FN400, and LPC. We hypothesized that the PA bouts result in improved behavioral outcomes in inhibitory control with associated decreases in P3 amplitude and latency similar to findings in young adults [30]. We also hypothesized improved episodic memory accompanied by an increase in LPC amplitude and reduced FN400 amplitude for PA conditions suggesting a shift for greater memory dependence for recollection compared to familiarity.

## 2. Materials and Methods

### 2.1. Participants

School-age children were recruited from central North Carolina communities through flyers and emails. Interested participants (*n* = 45; final analyses were performed on *n* = 36; see Statistical Analysis sectionSection 2.6 for exclusion reasons) and legal guardians provided informed consent and assent via a digital signature using an online Qualtrics survey (Qualtrics, Provo, UT, USA) in accordance with the Institutional Review Board (IRB) of the University of North Carolina at Greensboro. Legal guardians received additional electronic questionnaires to complete on behalf of the participant prior to the first visit to the laboratory. These surveys included health history and demographics, puberty status utilizing the Tanner Staging System [43], socioeconomic status (SES) according to the level of the mothers’ education, and the Physical Activity Readiness Questionnaire (PAR-Q) [44]. Based on these surveys, participants included in the study had normal or corrected-to-normal vision based on the minimal 20/20 standard, did not indicate potential health risks from engaging in moderate or high-intensity PA (based on PAR-Q), scored above 80 on the IQ measure, and indicated English as their primary language. No participants were excluded based on these inclusion criteria. All interested participants were invited to the laboratory for further testing. On successful completion of the study, participants were compensated at USD10/h while legal guardians were compensated at USD10 for each laboratory visit.

### 2.2. Fitness Assessment

Each participant was fitted with a Polar heart rate (HR) monitor and measurements of height and weight were recorded (stadiometer and a Tanita WB-300 Plus digital scale). Body mass index (BMI) was calculated as the weight divided by the square of the height (i.e., kg/m^2^). For the cardiovascular fitness assessment, a modified Balke protocol [27] was used while participants ran on a motor-driven treadmill and maximal aerobic capacity (VO_2_ peak) was measured using a computerized indirect calorimetry system (ParvoMedics True Max 2400 L). VO_2_ peak was established when children met at least two of the protocol criteria [27]. Relative peak oxygen consumption was expressed in milliliters of oxygen consumed per kilogram of body weight per minute (mL/kg/min).

### 2.3. Cognitive Tasks

#### 2.3.1. Flanker Task

Participants completed a modified version of a flanker task [45] to assess aspects of inhibitory control. The flanker task involved stimuli consisting of five yellow fish (3 cm tall) presented at the center of a blue screen using PsychoPy software (version 1.90.3) [46]. Each stimulus was presented for 350 ms with a variable inter-trial interval (ITI) of 1800 ms, 2000 ms, and 2200 ms to introduce temporal uncertainty. The flanker task required participants to pay attention to the center fish and respond in accordance with directionality (e.g., left thumb response if the center fish was pointing left and vise-versa) amidst either congruent (>>>>> or <<<<<) or incongruent (>><>> or <<><<) trials using a 4-button response pad (Current Designs Inc., Philadelphia, PA, USA). Task instructions emphasized response accuracy and response speed (i.e., “It is important that you respond as accurately as possible, but we also want you to respond quickly so please make sure you respond before the next set of fish appears on the screen”). Participants completed three blocks of 108 trials consisting of equiprobable congruent and incongruent trials. The trial order was randomized within each block. Prior to each experimental session, participants completed a practice block of 52 trials to familiarize themselves with the task requirements. Accuracy and reaction time (RT) measures were assessed for each trial condition (congruent, incongruent).

#### 2.3.2. Word Recognition Task

Participants completed a word recognition task. Words were selected from the MRC Psycholinguistic Database [47,48] based on the following criteria that matched for children of the age in the present study: number of letters (3–6), written frequency, concreteness, familiarity, and age of acquisition. Words were then assigned to nonoverlapping word lists. All word lists contained an equiprobable value of words that matched the above selection criteria. This was to ensure that each iteration of the word recognition task was equivalent in difficulty for developing children. The word recognition task included two phases. The first phase (i.e., encoding) required participants to memorize a list of 30 words presented on a computer screen, ignoring sequential presentation without making a response. Words were presented (3 cm tall, white Arial font) on a black screen for 2000 ms duration with a variable ITI of 4000 ms, 4500 ms, and 5000 ms. Following the encoding phase, the flanker task was performed that afforded a ~10-min delay prior to the recognition phase. The delayed recognition phase consisted of a random presentation of the 30 words from the study phase (i.e., old words) randomly intermixed with 30 new words. Stimulus duration was set at 2000 ms with a variable ITI of 3000 ms, 3500 ms, and 4000 ms to introduce temporal uncertainty. Participants were instructed to respond as quickly and accurately as possible with a button response to old and new words. Left and right response options for old and new word designations were counterbalanced across participants. For example, participant A required a left response for old words and a right response for new words, while participant B required a right response for old words and a left response for new words. Outcome variables were response accuracy and RT separately for old and new word trials.

### 2.4. EEG

#### 2.4.1. Recording

Electroencephalography (EEG) activity was recorded during both flanker and memory recall task performance from 64 Ag/AgCl electrode sites using a Neuroscan Quick-Cap (Compumedics Neuroscan, Charlotte, NC, USA) organized in accordance with the international 10-10 system [49]. Prior to recordings, electrodes were filled with conductive gel, and impedance was maintained below 10 kW. To monitor electrooculographic (EOG) eye movement, vertical (VEOG) and horizontal (HEOG) bipolar electrodes were placed above and below the left orbit and outer canthus of each eye. Online data were referenced to a midline electrode between Cz and CPz with Fz acting as the ground electrode. Using a Neuroscan SynAmps2 amplifier, online continuous data were digitized at a sampling rate of 1000 Hz, amplified 500 times with a DC to 70 Hz band pass filter to record desired neural activity, and a 60 Hz notch filter was applied to reduce powerline noise.

#### 2.4.2. Processing

The offline data was processed using MATLAB (R2021a) and in conjunction with the EEGLAB [50] and ERPLAB [51] toolbox plugins. EEG data were re-referenced to averaged mastoids (M1, M2). A high-pass filter was applied to remove low frequency artifacts, with a cutoff frequency of 0.1 Hz. Bad channels were cleaned and/or removed using artifact subspace reconstruction (ASR) [52,53]. For ASR, we used a cutoff threshold of 3 standard deviations. This setting helps identify and remove artifacts that deviate significantly from the clean EEG data. The window length was set to 0.5 s. This parameter determines the size of the time window over which the ASR algorithm calculates the signal subspace. A step size of 0.1 s was used. This setting defines the overlap between consecutive windows, allowing for a smoother transition and more accurate artifact removal. The maximum number of dimensions retained for the signal subspace was set to 10. This parameter limits the dimensionality of the subspace to ensure that only the most significant components are retained. ASR was applied on a channel-by-channel basis to account for variations in artifact distribution across different channels. Once bad channels were identified using ASR, they were temporarily removed from the dataset to prevent their noise from affecting subsequent ICA analyses. Following ICA eyeblink artifact removal (see below), bad channels were interpolated to ensure the integrity of the dataset, using spherical spline interpolation. This method estimates the signal at the bad channel by weighting the signals from surrounding channels based on their spatial proximity. Spherical spline interpolation is a widely accepted method in EEG preprocessing for reconstructing the signal at bad channels. It allows for the inclusion of the reconstructed channel in further analyses, thereby maintaining the overall channel configuration and spatial resolution of the EEG data. Eyeblink artifact was removed utilizing an automated independent component analysis (ICA) procedure. ICA decompositions were performed using the extended infomax algorithm to extract sub-Gaussian components with the default MATLAB implementation of this function. Subsequently, the eyeblink artifact components were identified using icablinkmetrics plugin [54] which is a time series correlation method comparing the raw VEOG data with distinct ICA activation waveforms. To ensure consistency and temporal alignment with raw VEOG artifacts in the continuous EEG data, any ICA components exhibiting high correlation (r = 0.8) with VEOG were removed. After the removal of the identified ICA components, the data were back projected, resulting in the restoration of the EEG signals without the rejected ICA components.

#### 2.4.3. ERPs

Stimulus-locked epochs were created for the flanker task (−200 ms to 1200 ms) and recognition memory task (−200 ms to 2000 ms) encompassing correct responses. Epochs were baseline corrected using prestimulus intervals (−200 ms to 0 ms) and low-pass filtered at 30 Hz. Individual epochs were rejected if a moving window peak-to-peak amplitude exceeded 100 µV (100 ms window width and 50 ms window step) evaluated at all midline sites. Grand average waveforms were created separately for each trial condition (i.e., congruent, incongruent; old, new) from remaining correct trials following cleaning procedures. P3 mean amplitude and fractional latency were evaluated between 400 ms to 800 ms at sites Cz, CPz, and Pz. For the word recognition task, difference waves were created from grand average waveforms (old correct–new correct). For old, new, and difference ERPs, the FN400 mean amplitude was evaluated between 300 ms to 500 ms and the LPC mean amplitude was evaluated between 500 ms to 900 ms at frontal (F3, Fz, F4), central (C3, Cz, C4) and parietal (P3, Pz, P4) sites.

### 2.5. Procedure

Using a within-participants crossover design, all participants attended the lab on three separate days (approximately one week between visits) and took part in three separate testing conditions—including a single bout of moderate-intensity cycling, seated rest, and HIIE—with the order of condition randomized across participants. Participants were instructed to avoid vigorous PA and to maintain typical daily behaviors (i.e., sleep, food and beverage consumption, and work/school activities) 24 h prior to testing. For each testing session, participants were fitted with an HR monitor and EEG cap followed by 9 min of the testing condition and then completion of cognitive tasks and EEG recordings in a quiet testing chamber. Prior to starting the cycling session, all participants completed an intelligence quotient (IQ) by a trained experimenter using the Weschsler Abbreviated Scale of Intelligence (WASI-II), an age-normed standardized assessment of cognitive ability, as cognition has been found to be sensitive to this factor [55]. Following the seated rest session, all participants completed the cardiorespiratory fitness assessment.

The HIIE session consisted of 30 s of in-place high-intensity calisthenics (>90% age-predicted HRmax) followed by 30 s of rest, repeated for 9 min. The type of calisthenics was performed in the following order for all participants: high knees, star jumps, butt kickers, high knees, jumping jacks, lateral hops, lunges, star jumps, and air squats. The current protocol was adapted for children who may be in a classroom environment with limited space for movement. The intent of the protocol was to provide a high-intensity exercise routine in a short amount of time that may be performed in a stationary location like next to a desk or in a designated space in a classroom. For the cycling condition, participants pedaled at a constant speed on a stationary bike at 70% age-predicted HRmax. For the seated rest condition, all participants watched an educational video (*“Join this Man on a Safari to Sculpt Animals in the Wild;”* National Geographic). HR and ratings of perceived exertion (RPE) were recorded by an experimenter every 30 s during experimental conditions (corresponding with time of intensity change for the HIIE condition) and at eight minutes following the cessation of each experimental condition.

### 2.6. Statistical Analysis

Final analyses were performed on thirty-six participants (*n* = 36). Participants were excluded if they did not come to the laboratory for any testing days (*n* = 7) or revealed poor performance on the flanker task (<40% accuracy; *n* = 2). Multiple imputation methods with 20 iterations were utilized to account for missing data from participants who only attended one (*n* = 2) or two sessions (*n* = 1; canceled because of COVID-19 closures), and ERP measures with no clean ERP trials to create a grand-average waveform for the recognition memory task (samples missing at random: FN400, *n* = 9; LPC, *n* = 8). An a priori power analysis was conducted using G*Power v3.1.9 [56] for sample size estimation in repeated measures (i.e., three repeated measures) within-factors design. Results from previous meta-analytic reports evaluating neurophysiological functioning in children following acute physical activity revealed a small (0.32) effect size [57,58]. Therefore, power analysis with an effect size of 0.32 (power = 0.8, and alpha = 0.05) revealed *n* = 24 as the projected sample size necessary to determine an effect. Thus, the present design appears adequately powered for statistical analyses of the neurophysiological data. Although a larger sample size could potentially increase the robustness of our findings, the within-participants design enhances the statistical power by reducing inter-subject variability. Each child served as their own control, allowing for a more precise estimation of the effects of physical activity on cognitive and neurophysiological outcomes. The repeated-measures ANOVA approach utilized in our analyses was well-suited for this design and was capable of detecting significant differences with the given sample size.

Analyses were conducted utilizing repeated-measures ANOVA with main effects and interactions reported using the Huynh–Feldt correction statistic for violations of sphericity and partial *ηp*^2^. Post hoc *t*-test comparisons included reporting of estimated effect size (Cohen’s *d*; small ≤ 0.2, medium = 0.5, and large ≥ 0.8 effect sizes) with false discovery rate correction [(individual *p* value rank/total number of comparisons) × (false discovery rate i.e., 0.15)]. Flanker response accuracy and RT were analyzed using a 3 (Mode: HIIE, cycling, rest) × 2 (Type: congruent, incongruent trials) model. Word recognition response accuracy and RT were analyzed using a 3 (Mode: HIIE, cycling, and rest) × 2 (Type: old, new) model. P3 was analyzed using a 3 (Mode: HIIE, cycling, rest) × 2 (Type: congruent, incongruent) × 3 (Site: Cz, CPz, Pz) model. FN400 and LPC were analyzed separately using the following models for different regions across the scalp and different waveform types. For the frontal region, separate 3 (Mode: HIIE, cycling, rest) × 3 (Site: F3, Fz, F4) models were used to analyze old, new, and difference waveforms. For the central region, separate 3 (Mode: HIIE, cycling, rest) × 3 (Site: C3, Cz, C4) models were used to analyze old, new, and difference waveforms. Lastly, for the parietal region, separate 3 (Mode: HIIE, cycling, rest) × 3 (Site: P3, Pz, P4) models were used to analyze old, new, and difference waveforms.

**Figure 1:**
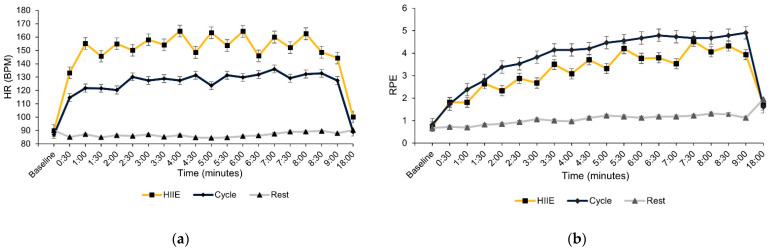
(**a**) Heart rate. (**b**) Ratings of perceived exertion (RPE). Note: HR reported as beats per minute (BPM), RPE measured using the Children’s OMNI-walk/run Scale of Perceived Exertion (category range, 0–10).

The means and standard deviation (±SD) are reported for demographics and fitness measures in Table 1. Preliminary analyses on HR and RPE were performed to determine intervention manipulation checks. Simple *t*-tests did not reveal any differences between the three conditions at baseline for HR [*t*’s (35) ≤ 1.44, *p*’s ≥ 0.15] and RPE [*t*’s (35) ≤ 0.71, *p*’s ≥ 0.48]. For the experimental conditions, HR and RPE were averaged across the 9 min period and compared with simple *t*-test comparisons. The mean HR during experimental sessions revealed a greater HR for the HIIE (147.2 ± 5.3 bpm) compared to cycling (126.7 ± 6.7 bpm) and rest [87.1 ± 2.5 bpm; *t*’s (35) ≥ 7.15, *p*’s ≤ 0.01], and greater HR for cycling compared to rest [*t* (35) = 5.15, *p* ≤ 0.01; see Figure 1a]. The mean RPE during each experimental session revealed a greater RPE for the cycling (4.1 ± 2.9) compared to HIIE (3.3 ± 3.3) and rest [1.1 ± 2.8; *t*’s (35) ≥ 2.20, *p*’s ≤ 0.03] and greater RPE for HIIE compared to rest [*t* (35) = 6.05, *p* ≤ 0.01; see Figure 1b]. A simple *t*-test comparison at 18 min (9 min post experimental conditions) did not reveal any significant difference for RPE [*t*’s (35) ≤ 0.94, *p*’s ≥ 0.35]. However, the mean HR remained elevated for the HIIE (100.26 ± 2.8 bpm) compared to both cycling (89.01 ± 2.5 bpm) and rest (90.10 ± 2.6 bpm), [*t*’s (35) ≥ 3.01, *p*’s ≤ 0.01].

## 3. Results

### 3.1. Cognitive Task Performance

**Figure 2:**
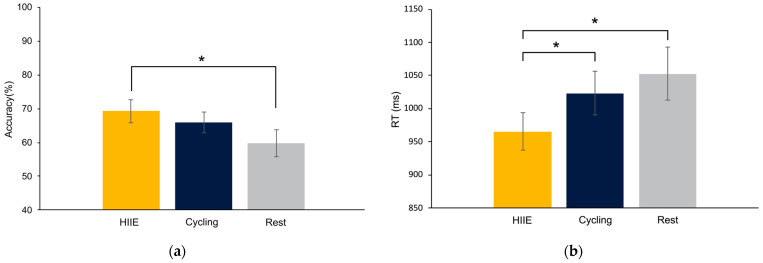
(**a**) Word recognition response accuracy for old words across high-intensity interval exercise (HIIE), moderate-intensity cycling, and seated rest conditions. (**b**) Word recognition mean reaction time (RT) for old words across HIIE, cycling, and rest conditions. RT is reported in milliseconds (ms). Significant differences are indicated by * *p* < 0.05.

The omnibus analysis for flanker performance (accuracy and RT) only revealed main effects of Type, [F’s (1,35) ≥ 33.85, *p*’s ≤ 0.01, *ηp*^2^’s ≥ 0.49], revealing greater accuracy and shorter RT for congruent trials (91.05 ± 1.50%; 550.91 ± 16.01 ms) compared to incongruent trials (87.53 ± 1.69%; 570.67 ± 15.96 ms). The omnibus analysis for word recognition accuracy revealed a main effect of Type [F (1,35) = 31.79, *p* ≤ 0.01, *ηp*^2^ = 0.47] that was superseded by a Mode × Type interaction, [F (1,35) = 4.32, *p* = 0.05, *ηp*^2^ = 0.11]. Decomposition of the interaction revealed greater response accuracy for old words in the HIIE condition (69.27 ± 3.36%) compared to the rest condition (59.83 ± 4.02%), *t* (35) = 2.13, *p* = 0.03, *d* = 0.38 (See Figure 2a). Further analysis for RT revealed a main effect of Mode [F (1,35) = 4.99, *p* ≤ 0.02, *ηp*^2^ = 0.12] revealing shorter RT for the HIIE (965.63 ± 28.48 ms) compared to rest (1052.69 ± 39.73 ms) and cycling (1023.29 ± 32.62 ms), [*t*’s (35) ≥ 2.19, *p*’s ≤ 0.03, *d*’s > 0.38; see Figure 2b]. Lastly, a main effect of Type [F (1,35) = 7.83, *p* ≤ 0.01, *ηp*^2^ = 0.18] revealed longer RT for new words (1041.32 ± 30.90 ms) compared to old words (988.87 ± 30.64 ms).

### 3.2. ERPs

#### 3.2.1. P3

The omnibus analysis for the P3 component showed a significant main effect of Type for mean amplitude [F (1,35) = 17.01, *p* ≤ 0.01, *ηp*^2^ = 0.33] revealing larger amplitude for incongruent trials (3.19 ± 0.36 µV) compared to congruent trials (2.55 ± 0.35 µV).

#### 3.2.2. FN400

Table 2 presents the significant main effects and interactions for the FN400 component. The key findings are summarized below with only significant main effects and interactions reported:

**Frontal Region (Sites F3, Fz, F4):** For old words, the amplitude at F4 (−4.42 ± 0.76 µV) was smaller compared to F3 (−5.74 ± 0.80 µV) and Fz (−5.37 ± 0.80 µV), [*t*’s (35) ≥ 2.97, *p*’s ≤ 0.01, *d*’s ≥ 0.53]. For new words, the amplitude at F4 (−3.99 ± 0.84 µV) was smaller compared to F3 (−4.83 ± 0.71 µV) and Fz (−4.96 ± 0.83 µV), [*t*’s (35) ≥ 2.06, *p*’s ≤ 0.04, *d*’s ≥ 0.36].

**Central Region (Sites C3, Cz, C4):** For old words, the amplitude at Cz (−5.21 ± 0.85 µV) was larger compared to C4 (−4.46 ± 0.70 µV), [*t* (35) = 1.96, *p* ≤ 0.05, *d* = 0.34].

**Figure 3:**
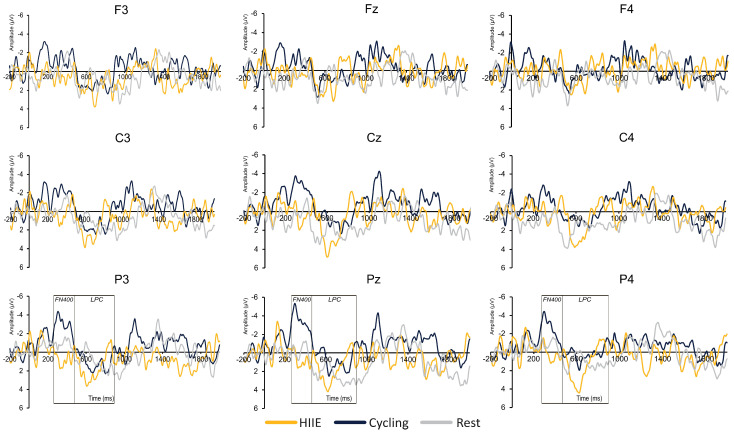
ERP difference waveforms (old − new words) across different regions and electrode sites. Each row represents regions with the top row representing the frontal region (F3, Fz, F4), middle row representing the central region (C3, Cz, C4), and the bottom row representing the parietal region (P3, Pz, P4). Waveforms are presented at each site for each condition: high-intensity interval exercise (HIIE), moderate-intensity cycling, and seated rest.

**Figure 4:**
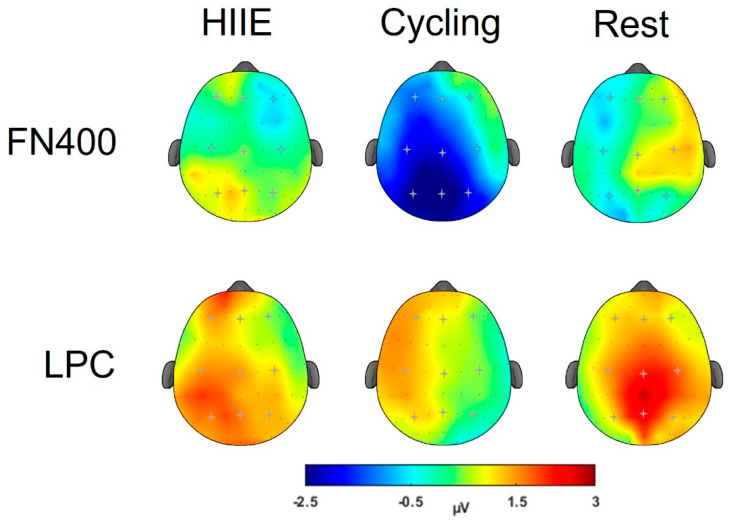
Topographic plots for FN400 and LPC difference waves (old − new words).

**Parietal Region (Sites P3, Pz, P4):** For old words, the amplitude at P4 (0.82 ± 0.61 µV) was larger compared to Pz (−0.74 ± 0.88 µV) and P3 (−1.28 ± 0.76 µV), [*t*’s (35) ≥ 3.61, *p*’s ≤ 0.01, *d*’s ≥ 0.62]. For new words, the amplitude at P4 (1.50 ± 0.72 µV) was larger compared to Pz (−0.14 ± 0.80 µV) and P3 (−0.26 ± 0.71 µV), [*t*’s (35) ≥ 3.81, *p*’s ≤ 0.01, *d*’s ≥ 0.65]. For the difference wave, the amplitude at the parietal region was larger for the cycling condition (−2.49 ± 0.84 µV) compared to HIIE (0.50 ± 0.68 µV) and rest (−0.30 ± 0.80 µV), [*t*’s (35) ≥ 3.55, *p*’s ≤ 0.05, *d*’s ≥ 0.34; see Figure 3 and Figure 4].

#### 3.2.3. LPC

**Figure 5:**
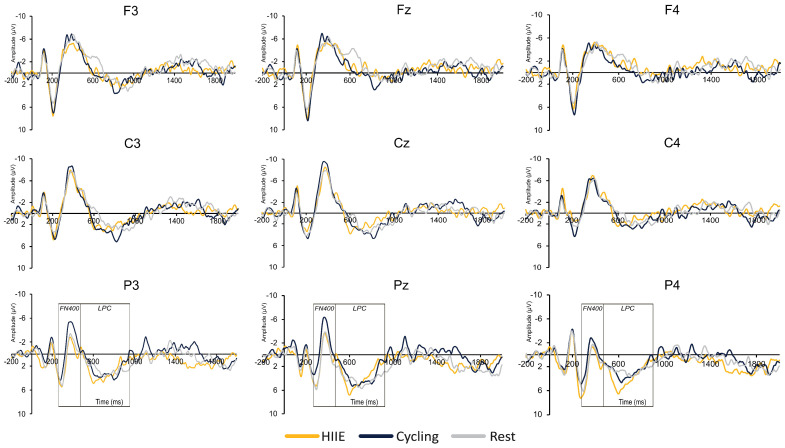
ERP old word waveforms across different regions and electrode sites. Each row represents regions with the top row representing the frontal region (F3, Fz, F4), middle row representing the central region (C3, Cz, C4), and the bottom row representing the parietal region (P3, Pz, P4). Waveforms are presented at each site for each condition: high-intensity interval exercise (HIIE), moderate-intensity cycling, and seated rest.

Table 3 presents the significant main effects and interactions for the LPC component. The key findings are summarized below with only significant main effects and interactions reported: 

**Frontal Region (Sites F3, Fz, F4):** For old words, larger amplitude was observed for cycling (0.27 ± 0.53 µV) compared to the rest condition (−1.82 ± 0.76 µV), [*t* (35) = 3.42, *p* ≤ 0.01, *d* = 0.57; see Figure 5]. In addition, smaller amplitude for old words was observed at F3 (−0.14 ± 0.50 µV) compared to Fz (−1.06 ± 0.54 µV) and F4 (−1.18 ± 0.47 µV), [*t*’s (35) ≥ 3.56, *p*’s ≤ 0.01, *d*’s ≥ 0.69].

**Figure 6:**
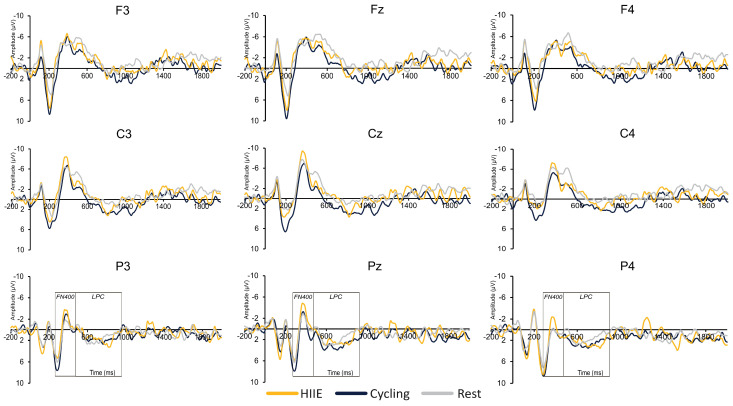
ERP new word waveforms across different regions and electrode sites. Each row represents regions with the top row representing the frontal region (F3, Fz, F4), middle row representing the central region (C3, Cz, C4), and the bottom row representing the parietal region (P3, Pz, P4). Waveforms are presented at each site for each condition: high-intensity interval exercise (HIIE), moderate-intensity cycling, and seated rest.

**Central Region (Sites C3, Cz, C4):** For old words, results revealed smaller amplitude at C4 (0.98 ± 0.43 µV) compared to Cz (2.02 ± 0.54 µV) and C3 (1.66 ± 0.49 µV), [*t*’s (35) ≥ 2.15, *p*’s ≤ 0.03, *d*’s ≥ 0.38]. For new words, results revealed larger LPC amplitude for the cycling condition (1.05 ± 0.64 µV) compared to the rest condition (−1.35 ± 0.72 µV), [*t* (35) = 3.20, *p* ≤ 0.01, *d* = 0.54; see Figure 6].

**Parietal Region (Sites P3, Pz, P4):** For old words, larger amplitude was observed at Pz (4.21 ± 0.50 µV) compared to P3 (2.95 ± 0.46 µV) and P4 (3.19 ± 0.40 µV), [*t*’s (35) ≥ 3.18, *p*’s ≤ 0.01, *d*’s ≥ 0.57]. For new words, smaller amplitude was observed at P3 (1.37 ± 0.35 µV) compared to Pz (2.09 ± 0.45 µV) and P4 (1.94 ± 0.46 µV), [*t*’s (35) ≥ 2.02, *p*’s ≤ 0.05, *d*’s ≥ 0.34]. For difference waves, the main effect of Site revealed a larger amplitude only at Pz (2.01 ± 0.47 µV) compared to P4 (1.14 ± 0.45 µV), [t (35) = 2.85, *p* ≤ 0.01, d = 0.49; see Figure 4].

## 4. Discussion

The present study evaluated the effects of short 9 min bouts of PA on inhibitory control and episodic memory in children. The flanker task results did not reveal any differences between conditions for behavioral performance and P3 suggesting a nuanced relationship between short bouts of PA and attentional processing. The word recognition task indicated that the HIIE condition enhanced accuracy in recalling previously encountered words compared to the rest condition. This enhancement was coupled with faster RT relative to both rest and cycling conditions, underscoring the potential of HIIE in improving episodic memory. The FN400 and LPC results may suggest differential processing strategies induced by the exercise modalities, with potential implications for memory retrieval processes.

The behavior and P3 results from the flanker task are in line with similar young adult research revealing no change in flanker performance and P3 after 9 min for both calisthenics and aerobic HIIE [59]. However, it should be noted that contrasting findings in young adults reveal improvements in behavior after 9 and 33 min of aerobic HIIE [32,60] with reduced P3 following 9 min [60]. In children, inhibitory control performance improves following 5 min bouts at low-, moderate-, and high-intensity exercise [61]. The absence of similar enhancements in the present findings could be attributed to several factors. First, the duration and intensity of the exercise bout may not have been sufficient to elicit measurable changes in inhibitory control in children. Previous studies suggest a threshold effect where certain intensity and duration are required to observe cognitive benefits [8]. This is warranted by a recent meta-analysis regarding P3 changes that revealed exercise duration between 11 and 30 min yielded small to medium effects on P3 amplitude (ES = 0.25 and 0.51) compared to exercise duration shorter than 10 min (ES = 0.024) [57]. Additionally, developmental differences between children and adults in neural maturation and cognitive processing capacities might influence how exercise impacts cognitive functions. Further research should explore the optimal exercise parameters and underlying mechanisms that facilitate cognitive improvements in children. Our study highlights the complexity of exercise-cognition interactions and underscores the need for age-specific exercise recommendations to enhance cognitive health.

Unlike inhibitory control, the behavioral results from the word recognition task revealed enhanced episodic memory (i.e., improved recognition), particularly for old words following HIIE. Other memory studies have also demonstrated improved memory performance [24,60], as well as enhanced primacy accuracy (i.e., recall of first 10 words) in children following moderate-intensity PA [12]. Although these HIIE findings are novel, it should be noted that the moderate-intensity cycling condition in the present study revealed no differences in behavioral performance despite replicating prior work of moderate-intensity PA but for a shorter duration. A possible explanation is that short bouts of moderate-intensity cycling conditions may not have been long enough to elicit behavioral differences. This is supported by a meta-analysis revealing that exercise time lower than 20 min may not improve episodic memory suggesting exercise duration may have a pivotal role in influencing episodic memory [33]. 

Regarding the FN400 and LPC results, only the cycling condition exhibited larger FN400 and LPC amplitude. These results suggest that cycling conditions upregulated familiarity and recollection-based recognition memory processes [37]. While Loprinzi and team (2019) proposed that complex movements, similar to the calisthenics performed in the present study, would have a greater effect on episodic memory as they increase cerebral blood flow and cortical excitability [33,61]. However, their analysis revealed that cycling-based exercises had a greater effect on episodic memory [33]. The authors interpreted that novice participants may perceive low- to moderate-intensity cycling as high-intensity PA which may have resulted in larger FN400 and LPC amplitudes following moderate-intensity cycling in the current findings. Furthermore, the ERP findings for the cycling condition did not align with the improvements in memory performance following the HIIE condition. One potential reason for these discrepancies may lie in the intensity-specific effects on episodic memory. High-intensity exercise may favor behavioral outcomes for episodic memory over moderate-intensity PA [33]. For instance, Winter et al. [62] demonstrated improved retention of word pairs following high-intensity exercise (i.e., two sprints of 3 min) compared to moderate-intensity running. These data support our behavior findings for HIIE and not the cycling condition. Together, these findings may provide preliminary evidence for behavioral and neural underpinnings that support memory improvements following short bouts of PA.

A proposed mechanism for the observed changes in memory and ERPs may be the activation of the nucleus of the tractus solitaries (NTS) and locus coeruleus (LC) following acute bouts of PA [63]. Research suggests that HIIE upregulates the release of neurotransmitters within these regions including norepinephrine, glutamate, dopamine, serotonin, and acetylcholine [64]. Moreover, previous work indicated changes in the release of neurotransmitters within minutes of exercise onset [63,64,65,66,67,68], and upregulation of AMPA (α-amino-3-hydroxyl-5-methyl-4-isoxazole-propionate) and NMDA (N-methyl-D-aspartate) receptors [69,70,71], in strengthening synaptic connections and potentiation facilitating the conscious recollection of words stored in the hippocampus [63]. These mechanisms may collectively contribute to the improved memory performance observed following a short bout of HIIE.

The present study offers valuable insights into the effects of short bouts of PA on episodic memory; however, several limitations should be noted. First, baseline cognitive measurements were not assessed across conditions, which restricts our ability to conclude any potential results in the PA and memory relationship [72]. A recent review paper by Ishihara et al. [73] emphasized the significance of baseline assessments (within-subjects design) in moderating the beneficial effects of acute PA on executive function. However, one of the strengths of the present study was employing a within-participants study design controlling for individual differences across conditions making it more suitable for investigating acute PA effects on cognition. Second, the present study did not collect measures of PA preference to determine individual differences that may influence cognitive outcomes [33]. Third, prior research suggests that intra-individual differences in psychological states (such as high- and low-affect) may mediate cognitive outcomes following acute PA [74]. Future research should consider psychological mental states as a potential mediator in evaluating the effects of PA on cognitive outcomes. Finally, the current sample was predominantly White or Caucasian (80%), which limits the generalizability of our findings to a more diverse population.

## 5. Conclusions

In conclusion, this study assessed the impact of short bouts of PA on inhibitory control and episodic memory in children. These findings demonstrate that engaging in a short bout of HIIE may enhance the recollection of words among children. Additionally, moderate-intensity cycling did not reveal any behavioral outcomes yet appeared to prompt neurocognitive familiarity and recollection-based recognition memory processes. These findings underscore the potential benefits of integrating feasible short bouts of PA into a classroom setting to enhance memory performance among children.

## Figures and Tables

**Table 1:** Means (±SD) for demographics and fitness measures.

Measures	Participants
N (females)	36 (18)
Age (years)	10.1 ± 1.1
Puberty Timing	1.9 ± 0.9
BMI	19.2 ± 3.6
Fitness (mL/kg/min)	37.6 ± 7.7
Fitness percentile (%)	42.2 ± 32.3
IQ	103.5 ± 11.7
Maternal Education	
Advanced degree	14
Bachelor’s degree	20
Some college	1
Hispanic or Latino	
Yes	7
No	28
Race	
White or Caucasian	25
Black or African	3
Mixed	4
Asian	2
Asian, White, or Caucasian	1
Not reported	1

Note: Two participants did not complete the maximal fitness assessment. Puberty timing was measured using the Tanner Staging System with “prepubescent” score between 1 and 2. BMI calculated as weight divided by square of height (i.e., kg/m^2^). IQ = intelligent quotient measured with the Weschsler Abbreviated Scale of Intelligence second edition (WASI-II).

**Table 2:** Summary of repeated measures of ANOVA for FN400.

Model	F	df1/df2	*p*	*ηp* ^2^
**FN400**				
**Frontal Region (F3, Fz, F4)**				
Site (Old)	7.58	1,35	<0.01	0.18
Site (New)	5.17	1,35	<0.02	0.13
**Central Region (C3, Cz, C4)**				
Site (Old)	6.43	1,35	<0.01	0.15
**Parietal Region (P3, Pz, P4)**				
Site (Old)	13.15	1,35	<0.01	0.27
Site (New)	12.10	1,35	<0.01	0.25
Mode (Difference)	5.24	1,35	<0.01	0.13

Note: Significant main effects and interactions are highlighted in this table. “Site” refers to the factor associated with electrode positions on the scalp (Frontal, Central, Parietal). “Mode” refers to the factor associated with condition including HIIE, moderate-intensity cycling, and seated rest. “Old” and “New” refer to the conditions of the word recognition task. “Difference” the calculated ERP difference waveform between old and new word conditions.

**Table 3:** Summary of repeated measures of ANOVA for LPC.

Model	F	df1/df2	*p*	*ηp* ^2^
**LPC**				
**Frontal Region (F3, Fz, F4)**				
Mode (Old)	5.19	1,35	<0.04	0.13
Site (Old)	13.94	1,35	<0.01	0.28
**Central Region (C3, Cz, C4)**				
Site (Old)	7.13	1,35	<0.01	0.17
Mode (New)	4.54	1,35	<0.01	0.11
**Parietal Region (P3, Pz, P4)**				
Site (Old)	11.92	1,35	<0.01	0.25
Site (New)	4.99	1,35	<0.01	0.12
Site (Difference)	4.54	1,35	<0.05	0.11

Note: Significant main effects and interactions are highlighted in this table. “Site” refers to the factor associated with electrode positions on the scalp (Frontal, Central, Parietal). “Mode” refers to the factor associated with condition including HIIE, moderate-intensity cycling, and seated rest. “Old” and “New” refer to the conditions of the word recognition task. “Difference” the calculated ERP difference waveform between old and new word conditions.

## Data Availability

The data presented in this study are available on request from the corresponding author due to privacy and ethical considerations. Additionally, the study is ongoing, and sharing the data at this stage might compromise the integrity of the research. Therefore, access is restricted to protect participant privacy and comply with ethical guidelines.

## References

[1] Gustafsson M. (2021). Pandemic-Related Disruptions to Schooling and Impacts on Learning Proficiency Indicators: A Focus on the Early Grades.

[2] McArthur B.A., Racine N., Browne D., McDonald S., Tough S., Madigan S. (2021). Recreational screen time before and during COVID-19 in school-aged children. Acta Paediatr..

[3] Neville R.D., Lakes K.D., Hopkins W.G., Tarantino G., Draper C.E., Beck R., Madigan S. (2022). Global Changes in Child and Adolescent Physical Activity During the COVID-19 Pandemic: A Systematic Review and Meta-analysis. JAMA Pediatr..

[4] Kohl H.W., Craig C.L., Lambert E.V., Inoue S., Alkandari J.R., Leetongin G., Kahlmeier S. (2012). The pandemic of physical inactivity: Global action for public health. Lancet.

[5] World Health Organization (WHO) (2018). Global Action Plan on Physical Activity 2018–2030. https://apps.who.int/iris/handle/10665/353808.

[6] Hillman C.H., Logan N., Shigeta T. (2019). A Review of Acute Physical Activity Effects on Brain and Cognition in Children. Transl. J. Am. Coll. Sports Med..

[7] Booth J.N., Chesham R.A., Brooks N.E., Gorely T., Moran C.N. (2020). A citizen science study of short physical activity breaks at school: Improvements in cognition and wellbeing with self-paced activity. BMC Med..

[8] Chang Y.K., Labban J.D., Gapin J.I., Etnier J.L. (2012). The effects of acute exercise on cognitive performance: A meta-analysis. Brain Res..

[9] de Greeff J.W., Bosker R.J., Oosterlaan J., Visscher C., Hartman E. (2018). Effects of physical activity on executive functions, attention and academic performance in preadolescent children: A meta-analysis. J. Sci. Med. Sport.

[10] Drollette E.S., Shishido T., Pontifex M.B., Hillman C.H. (2012). Maintenance of cognitive control during and after walking in preadolescent children. Med. Sci. Sports Exerc..

[11] Drollette E.S., Scudder M.R., Raine L.B., Moore R.D., Saliba B.J., Pontifex M.B., Hillman C.H. (2014). Acute exercise facilitates brain function and cognition in children who need it most: An ERP study of individual differences in inhibitory control capacity. Dev. Cogn. Neurosci..

[12] Drollette E.S., Hillman C.H. (2020). Walking effects on memory in children: Implications for individual differences in BMI. Ment. Health Phys. Act..

[13] Ludyga S., Gerber M., Brand S., Holsboer-Trachsler E., Pühse U. (2016). Acute effects of moderate aerobic exercise on specific aspects of executive function in different age and fitness groups: A meta-analysis: Moderate exercise and executive function. Psychophysiology.

[14] Tomporowski P.D., Davis C.L., Miller P.H., Naglieri J.A. (2008). Exercise and Children’s Intelligence, Cognition, and Academic Achievement. Educ. Psychol. Rev..

[15] Tomporowski P.D., McCullick B., Pendleton D.M., Pesce C. (2015). Exercise and children’s cognition: The role of exercise characteristics and a place for metacognition. J. Sport Health Sci..

[16] Diamond A., Barnett W.S., Thomas J., Munro S. (2007). Preschool Program Improves Cognitive Control. Science.

[17] Diamond A. (2015). The Cognitive Benefits of Exercise in Youth. Curr. Sports Med. Rep..

[18] St Clair-Thompson H.L., Gathercole S.E. (2006). Executive functions and achievements in school: Shifting, updating, inhibition, and working memory. Q. J. Exp. Psychol..

[19] Botvinick M.M., Carter C.S., Braver T.S., Barch D.M., Cohen J.D. (2001). Conflict Monitoring and Cognitive Control. Psychol. Rev..

[20] Donnelly J.E., Hillman C.H., Castelli D., Etnier J.L., Lee S., Tomporowski P.D., Lambourne K., Szabo-Reed A.N. (2016). Physical Activity, Fitness, Cognitive Function, and Academic Achievement in Children: A Systematic Review. Med. Sci. Sports Exerc..

[21] Hillman C.H., Pontifex M.B., Raine L.B., Castelli D.M., Hall E.E., Kramer A.F. (2009). The effect of acute treadmill walking on cognitive control and academic achievement in preadolescent children. Neuroscience.

[22] Lambourne K., Tomporowski P.D. (2010). The effect of exercise-induced arousal on cognitive task performance: A meta-regression analysis. Brain Res..

[23] Tulving E. (1993). What Is Episodic Memory?. Curr. Dir. Psychol. Sci..

[24] Etnier J.L., Labban J.D., Piepmeier A., Davis M.E., Henning D.A. (2014). Effects of an Acute Bout of Exercise on Memory in 6th Grade Children. Pediatr. Exerc. Sci..

[25] Mavilidi M.F., Okely A.D., Chandler P., Paas F. (2016). Infusing Physical Activities into the Classroom: Effects on Preschool Children’s Geography Learning. Mind Brain Educ..

[26] Pesce C., Crova C., Cereatti L., Casella R., Bellucci M. (2009). Physical activity and mental performance in preadolescents: Effects of acute exercise on free-recall memory. Ment. Health Phys. Act..

[27] American College of Sports Medicine (2022). ACSM’s Guidelines for Exercise Testing and Prescription.

[28] Weston K.S., Wisløff U., Coombes J.S. (2014). High-intensity interval training in patients with lifestyle-induced cardiometabolic disease: A systematic review and meta-analysis. Br. J. Sports Med..

[29] Alves C.R.R., Tessaro V.H., Teixeira L.A.C., Murakava K., Roschel H., Gualano B., Takito M.Y. (2014). Influence of Acute High-Intensity Aerobic Interval Exercise Bout on Selective Attention and Short-Term Memory Tasks. Percept. Mot. Skills.

[30] Kao S.C., Westfall D.R., Soneson J., Gurd B., Hillman C.H. (2017). Comparison of the acute effects of high-intensity interval training and continuous aerobic walking on inhibitory control. Psychophysiology.

[31] Lambrick D., Stoner L., Grigg R., Faulkner J. (2016). Effects of continuous and intermittent exercise on executive function in children aged 8-10 years: Acute exercise and executive function. Psychophysiology.

[32] Tsukamoto H., Suga T., Takenaka S., Tanaka D., Takeuchi T., Hamaoka T., Isaka T., Hashimoto T. (2016). Greater impact of acute high-intensity interval exercise on post-exercise executive function compared to moderate-intensity continuous exercise. Physiol. Behav..

[33] Loprinzi P.D., Blough J., Crawford L., Ryu S., Zou L., Li H. (2019). The Temporal Effects of Acute Exercise on Episodic Memory Function: Systematic Review with Meta-Analysis. Brain Sci..

[34] Polich J. (2007). Updating P300: An integrative theory of P3a and P3b. Clin. Neurophysiol..

[35] Polich J. (2012). Neuropsychology of P300. The Oxford Handbook of Event-Related Potential Components.

[36] Pontifex M.B., Saliba B.J., Raine L.B., Picchietti D.L., Hillman C.H. (2013). Exercise Improves Behavioral, Neurocognitive, and Scholastic Performance in Children with Attention-Deficit/Hyperactivity Disorder. J. Pediatr..

[37] Rugg M.D., Curran T. (2007). Event-related potentials and recognition memory. Trends Cogn. Sci..

[38] Curran T. (2000). Brain potentials of recollection and familiarity. Mem. Cognit..

[39] Yonelinas A.P. (2002). The Nature of Recollection and Familiarity: A Review of 30 Years of Research. J. Mem. Lang..

[40] Leynes P.A., Bruett H., Krizan J., Veloso A. (2017). What psychological process is reflected in the FN400 event-related potential component?. Brain Cogn..

[41] Egan C.A., Webster C.A., Beets M.W., Weaver R.G., Russ L., Michael D., Nesbitt D., Orendorff K.L. (2019). Sedentary Time and Behavior during School: A Systematic Review and Meta-Analysis. Am. J. Health Educ..

[42] Yli-Piipari S., Kulmala J.S., Jaakkola T., Hakonen H., Fish J.C., Tammelin T. (2016). Objectively Measured School Day Physical Activity Among Elementary Students in the United States and Finland. J. Phys. Act. Health.

[43] Tanner J.M. (1962). Growth at Adolescence. Med. J. Aust..

[44] Thomas S., Reading J., Shephard R.J. (1992). Revision of the Physical Activity Readiness Questionnaire (PAR-Q). Can. J. Sport. Sci..

[45] Eriksen B.A., Eriksen C.W. (1974). Effects of noise letters upon the identification of a target letter in a nonsearch task. Percept. Psychophys..

[46] Peirce J., Gray J.R., Simpson S., MacAskill M., Höchenberger R., Sogo H., Kastman E., Lindeløv J.K. (2019). Experiments in behavior made easy. Behav. Res. Methods.

[47] Coltheart M. (1981). The MRC Psycholinguistic Database. Q. J. Exp. Psychol. Sect. A..

[48] Wilson M. (1988). MRC psycholinguistic database: Machine usable dictionary. Behav. Res. Methods Instrum. Comput..

[49] Chatrian G.E., Lettich E., Nelson P.L. (1985). Ten Percent Electrode System for Topographic Studies of Spontaneous and Evoked EEG Activities. Am. J. EEG Technol..

[50] Delorme A., Makeig S. (2004). EEGLAB: An open source toolbox for analysis of single-trial EEG dynamics. J. Neurosci. Methods.

[51] Lopez-Calderon J., Luck S.J. (2014). ERPLAB: An open-source toolbox for the analysis of event-related potentials. Front. Hum. Neurosci..

[52] Chang C.Y., Hsu S.H., Pion-Tonachini L., Jung T.P. Evaluation of Artifact Subspace Reconstruction for Automatic EEG Artifact Removal. Proceedings of the 2018 40th Annual International Conference of the IEEE Engineering in Medicine and Biology Society (EMBC).

[53] Kothe C.A., Makeig S. (2013). BCILAB: A platform for brain–computer interface development. J. Neural Eng..

[54] Pontifex M.B., Miskovic V., Laszlo S. (2017). Evaluating the efficacy of fully automated approaches for the selection of eyeblink ICA components. Psychophysiology.

[55] Davies P.L., Segalowitz S.J., Gavin W.J. (2004). Development of Response-Monitoring ERPs in 7- to 25-Year-Olds. Dev. Neuropsychol..

[56] Faul F., Erdfelder E., Lang A.-G., Buchner A. (2007). G*Power 3: A flexible statistical power analysis program for the social, behavioral, and biomedical sciences. Behav. Res. Methods.

[57] Kao S.-C., Chen F.-T., Moreau D., Drollette E.S., Amireault S., Chu C.-H., Chang Y.K. (2022). Acute effects of exercise engagement on neurocognitive function: A systematic review and meta-analysis on P3 amplitude and latency. Int. Rev. Sport. Exerc. Psychol..

[58] Meijer A., Königs M., Vermeulen G.T., Visscher C., Bosker R.J., Hartman E., Oosterlaan J. (2020). The effects of physical activity on brain structure and neurophysiological functioning in children: A systematic review and meta-analysis. Dev. Cogn. Neurosci..

[59] Drollette E.S., Johnson M.N., Meadows C.C. (2022). No Change in Inhibitory Control or P3 Following Different High-Intensity Interval Exercise Modalities. Brain Sci..

[60] Kao S.C., Drollette E.S., Ritondale J.P., Khan N., Hillman C.H. (2018). The acute effects of high-intensity interval training and moderate-intensity continuous exercise on declarative memory and inhibitory control. Psychol. Sport. Exerc..

[61] Gur R.C., Jaggi J.L., Ragland J.D., Resnick S.M., Shtasel D., Muenz L., Gur R.E. (1993). Effects of Memory Processing on Regional Brain Activation: Cerebral Blood Flow in Normal Subjects. Int. J. Neurosci..

[62] Winter B., Breitenstein C., Mooren F.C., Voelker K., Fobker M., Lechtermann A., Krueger K., Fromme A., Korsukewitz C., Floel A. (2007). High impact running improves learning. Neurobiol. Learn. Mem..

[63] Loprinzi P.D., Ponce P., Frith E. (2018). Hypothesized mechanisms through which acute exercise influences episodic memory. Acta Physiol. Int..

[64] Basso J.C., Suzuki W.A. (2017). The Effects of Acute Exercise on Mood, Cognition, Neurophysiology, and Neurochemical Pathways: A Review. Brain Plast..

[65] Chowdhury R., Guitart-Masip M., Bunzeck N., Dolan R.J., Düzel E. (2012). Dopamine Modulates Episodic Memory Persistence in Old Age. J. Neurosci..

[66] Hasselmo M.E. (2006). The Role of Acetylcholine in Learning and Memory. Curr. Opin. Neurobiol..

[67] Mlinar B., Stocca G., Corradetti R. (2015). Endogenous serotonin facilitates hippocampal long-term potentiation at CA3/CA1 synapses. J. Neural Transm..

[68] Stanton P.K., Sarvey J.M. (1985). Depletion of norepinephrine, but not serotonin, reduces long-term potentiation in the dentate gyrus of rat hippocampal slices. J. Neurosci. Off. J. Soc. Neurosci..

[69] Real C.C., Ferreira A.F.B., Hernandes M.S., Britto L.R.G., Pires R.S. (2010). Exercise-induced plasticity of AMPA-type glutamate receptor subunits in the rat brain. Brain Res..

[70] VanLeeuwen J.E., Petzinger G.M., Walsh J.P., Akopian G.K., Vuckovic M., Jakowec M.W. (2010). Altered AMPA receptor expression with treadmill exercise in the 1-methyl-4-phenyl-1,2,3,6-tetrahydropyridine-lesioned mouse model of basal ganglia injury. J. Neurosci. Res..

[71] Yu Q., Li X., Wang J., Li Y. (2013). Effect of exercise training on long-term potentiation and NMDA receptor channels in rats with cerebral infarction. Exp. Ther. Med..

[72] Loprinzi P.D., Loenneke J.P., Storm B.C. (2021). Effects of acute aerobic and resistance exercise on episodic memory function. Q. J. Exp. Psychol..

[73] Ishihara T., Drollette E.S., Ludyga S., Hillman C.H., Kamijo K. (2020). Baseline Cognitive Performance Moderates the Effects of Physical Activity on Executive Functions in Children. J. Clin. Med..

[74] Johnson M.N., Maher J.P., Meadows C.C., Bittel K.M., Hevel D.J., Drollette E.S. (2022). Positive affect moderates inhibitory control and positive affect following a single bout of self-select aerobic exercise. Psychol. Sport Exerc..

